# The Ly49 Gene Family. A Brief Guide to the Nomenclature, Genetics, and Role in Intracellular Infection

**DOI:** 10.3389/fimmu.2013.00090

**Published:** 2013-04-16

**Authors:** Alan Rowe Schenkel, Luke C. Kingry, Richard A. Slayden

**Affiliations:** ^1^Department of Microbiology, Immunology and Pathology, Colorado State UniversityFort Collins, CO, USA; ^2^Division of Vector Borne Diseases, Centers for Disease Control and PreventionFort Collins, CO, USA

**Keywords:** Ly49, klra, natural killer lymphocytes, myeloid cells

## Abstract

Understanding the Ly49 gene family can be challenging in terms of nomenclature and genetic organization. The Ly49 gene family has two major gene nomenclature systems, Ly49 and Killer Cell Lectin-like Receptor subfamily A (klra). Mice from different strains have varying numbers of these genes with strain specific allelic variants, duplications, deletions, and pseudogene sequences. Some members activate NK lymphocytes, invariant NKT (iNKT) lymphocytes and γδ T lymphocytes while others inhibit killing activity. One family member, Ly49Q, is expressed only on myeloid cells and is not found on NK, iNKT, or γδ T cells. There is growing evidence that these receptors may regulate not just the immune response to viruses, but other intracellular pathogens as well. Thus, this review’s primary goal is to provide a guide for researchers first encountering the Ly49 gene family and a foundation for future studies on the role that these gene products play in the immune response, particularly the response to intracellular viral and bacterial pathogens.

## Introduction

The highly polymorphic Ly49 genes serve as a reminder that nature and evolution occasionally conspire to resist systematic classification schemes. The first gene in this family was identified as a “T lymphocyte activation marker” and became Ly49A (Chan and Takei, 1989; Yokoyama et al., 1989). However, expression of Ly49A is found primarily on Natural Killer (NK), invariant NK T (To et al., 2009), and NK1.1+ γδ T lymphocytes (Hara et al., 2001). On T lymphocytes, it is found only on a subset of CD3+ T cells which were also NK1.1− and/or DX5− (Ortaldo et al., 1998) and a subset of CD8+ Tregs (Kim et al., 2011). The first identified function of the Ly49 genes was as an inhibitor of NK lymphocyte killing of tumor cell lines that express MHC Class I. NK killing was inhibited by Ly49A recognition of H2-D^d^ on the tumor cells (Yokoyama and Seaman, 1993). It was subsequently found that some Ly49 genes inhibited killing but others activated killing, both via interactions with MHC Class I molecules and other molecules like murine cytomegalovirus m157 on the surface of target cells (Makrigiannis and Anderson, 2000). Discoveries of more genes in the family quickly revealed that there was significant genomic complexity in the gene family, with different strains of mice having varying numbers of gene segments, arising in part from duplication events. Several Ly49 genomic sequences are non-functional pseudogenes (Yokoyama and Seaman, 1993; Smith et al., 1994).

Both inhibitory and activating Ly49 have the same basic structure, with a stalk and a natural killer receptor domain (NKD). They exist as homodimers on the surface of the cell, and interact with ligands via the NKD structures. However, inhibitory and activating Ly49 signal differently to the cell upon recognition of their ligands. The inhibitory Ly49 genes have a conserved Immunoreceptor Tyrosine-based Inhibitory motif (ITIM) domain on their cytoplasmic tails (Belanger et al., 2008). Activating Ly49 genes have a transmembrane arginine residue and associate with the signal adapter proteins DAP10 and DAP12. DAP12 is important for signaling but the requirement of DAP10 for signaling is potentially minimal (Tassi et al., 2009).

Perhaps unsurprisingly, this gene family also highlights the differences and similarities between mice and humans. Unlike other mammals such as rodents and cattle, the human genome does not encode Ly49 genes (Dissen et al., 2008). Instead, humans have the Killer Immunoglobulin-like Receptors (KIR) fulfilling the same role, and, like the Ly49 genes, KIR are highly polymorphic with both activating and inhibitory receptors. The genes are not genetically homologous to the Ly49 genes, but rather have a parallel function (Middleton and Gonzelez, 2010), and do appear to have evolved from similar duplication events as those seen in the Ly49 genes (Martin et al., 2000). Accordingly, the functional role the Ly49 genes may serve as a model to understand how KIR participate in the immune responses of human NK cells.

## Genomics of the Ly49 Genes

**Figure 1:**
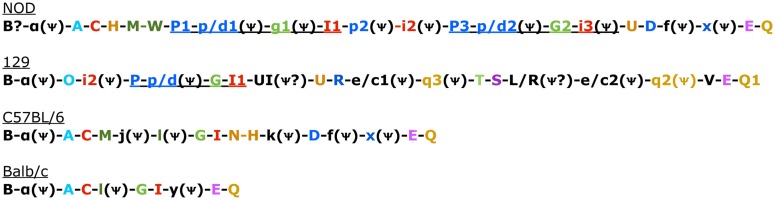
**Genomic Organization in the four known mouse strains**. Color coded to match Table 3 and Belanger et al. (2008). Uppercase gene is known to be expressed. Pseudogenes indicated with a psi symbol. Underlined sections indicate regions common in 129 and NOD mice that have also undergone duplication in NOD mice (Belanger et al., 2008).

Soon after cloning Ly49A, southern blots and analysis of mRNA transcripts revealed that there were several members of the Ly49 family (Yokoyama and Seaman, 1993; Smith et al., 1994), which has subsequently expanded to include approximately 20–30 members, including many pseudogenes (Table 1) (Makrigiannis and Anderson, 2000; Makrigiannis et al., 2001; Lavender and Kane, 2006; Kielczewska et al., 2007; Scarpellino et al., 2007; Belanger et al., 2008; Jonsson et al., 2010). This cluster was mapped to chromosome 6 (Yokoyama et al., 1990), mostly in tandem as shown in Figure 1, with the exception of Ly49B. Ly49B is also located on Chromosome 6 but not within the Ly49 cluster. Many gene segments, either fully functional genes or pseudogenes, are indicated by the nomenclature Ly49A-Ly49X and with the strain in superscript indicating the strain allelic variants, e.g., Ly49A^Balb/c^, Ly49A^C57BL/6^, or Ly49C^C57BL/6^. The first published usage of Killer Cell Lectin-like Receptor subfamily A (Klra) introduced a different systematic nomenclature system (Lee et al., 2001), which has been adopted by GenBank. GenBank also uses the Ly49 nomenclature and this can be confusing to researchers when they first encounter these two nomenclature systems. In our opinion, the use of the Ly49 with superscript strain nomenclature is the clearest and will be the system used by this review. In Figure 1 and Table 2, we will also use the shortened name, Ly49A^C57BL/6^ as A^B6^ for example. Similarly, we believe it would be useful to instead modify the klra nomenclature to klra1^C57BL/6^.

**Table 1:** **Combined nomenclature and ligands of the Ly49 genes**.

Ly49 GenBank	Ly49 NOD	Klra number	Role, strain, other notes	Other aliases, previous names or homologs	Ligands
	Ly49α		**Pseudogene** in Balb/c, NOD, C57BL/6 (B6), and 129 mice		N/A
Ly49A	Ly49a	Klra1	**Inhibitory**. C57/BL/6 (B6), NODShiLtJ (NOD), 129 and Balb/c	A1, Klra22, Ly49o^129^ (Ly49V)	H2-D^d^, H2-D^r^, H2-D^k^, H2-D^p^ (Makrigiannis and Anderson, 2000; Scarpellino et al., 2007; Jonsson et al., 2010). B6 strain: H2-L^d^, H2-D^d^ H2-K^b^ H2-D^b^ (Johansson et al., 2009)
Ly49B		Klra2	**Inhibitory** B6, 129, Balb/c. Two isoforms (splice variants). Unique locus 5′ from all other Ly49 genes	Klra30	Unknown
Ly49C	Ly49c	Klra3	**Inhibitory**. Balb/c, B6, and NOD, not 129; C/I are family of four genes; may be functional in NOD but has early stop codon	NK2.1, SW5E6 (5E6), Nk2, MGC123910	H2-D^d^, H2-D^k^, H2-D^b^, H2-K^b^, H2-K^d^, H2-L^d^, H2-K^k^ (Dimasi and Biassoni, 2005; Scarpellino et al., 2007; Johansson et al., 2009). Does not recognize CMV m157 protein (Kielczewska et al., 2007)
Ly49D	Ly49d	Klra4	**Activator**. B6 (2 isoforms) and NOD only	Chok, Klra32, Ly49r^129^	H2-D^d^, H2-D^r^, H2-D^sp2^ (Makrigiannis and Anderson, 2000)
Ly49E	Ly49e	Klra5	**Inhibitory** Balb/c, NOD, B6, and 129 (Aust et al., 2011)		Urokinase plasminogen activator (Van Den Broeck et al., 2008)
Ly49e/c		Klra26, Klra27	**Pseudogene** 129 has Ly49ec1^129^, Ly49ec2^129^ (Belanger et al., 2008)		N/A
Ly49F		Klra6	**Inhibitory** NOD, B6, and 129 mice		H2-D^d^ (Dimasi and Biassoni, 2005)
Ly49G	Ly49g1 = ψ; Ly49g2 = ITIM	Klra7	**Pseudogene (g1^NOD^); Inhibitory (g2^NOD^)** in NOD or **G** in B6, Balb/c, and 129 mice	LGL-1; MGC123448; MGC161317	NOD H2-K^d^ (weak) (Scarpellino et al., 2007); B6 strain H2-D^b^ (Johansson et al., 2009); 129 strain H2-D^d^, H2-D^k^, H2-K^d^ (Makrigiannis et al., 2001); Balb/c rat classical class I MHC molecule, RT1-A1^c^ (Lavender and Kane, 2006)
Ly49H	Ly49h	Klra8	**Activator**. B6 and NOD only	Cmv1, Ly49u^129^	CMV m157 (Arase et al., 2002; Kielczewska et al., 2007), H2-D^b^(Dimasi and Biassoni, 2005)
Ly49I	Ly49i1 = ITIM; Ly49i2 = ψ; Ly49i3 = ψ	Klra9	**Inhibitor +2 pseudogenes** in NOD; i1 and i2 ψ in 129 mice, one i in B6 and Balb/c		H2-K^b^, H2-K^d^ (Scarpellino et al., 2007); CMV m157 (Arase et al., 2002; Kielczewska et al., 2007); 129 strain H2-D^k^, H2-K^b^, H2-K^d^, H2-K^k^ (Makrigiannis et al., 2001) Ly49I^B6^ does not recognize CMV m157 protein (Kielczewska et al., 2007)
Ly49J		Klra10	**Pseudogene** in B6	Ly49i2	N/A
Ly49K		Klra11	**Pseudogene** B6 only		Unknown
Ly49L	See Ly49pd below	Klra12	**Pseudogene** Balb/c and B6 truncated	Ly49l1; Ly49l2; Ly49l3; Ly49l4; Ly49pd1 (see below), Ly49pd2 (see below)	N/A
Ly49l/r		Klra25	**Activator (putative)** 129 only (Makrigiannis et al., 2002), not known if expressed		Unknown
(Ly49p/d)	Ly49p/d1 = ψ; Ly49p/d2 = ψ		**Pseudogene** in NOD and 129 mice	Homolog of Ly49L	N/A
Ly49M	Ly49m	Klra13	**Activator**. B6 and NOD only;	MGC123449	Unknown
Ly49N		Klra14	**Activator**. Predicted gene, homology with Klra8, Klra21	Klra29; EG654449; OTTMUSG0000002 6959; Gm15858	Unknown
Ly49O		Klra15	**Inhibitor**. 129 (Makrigiannis et al., 1999) and C57L (Mehta et al., 2001)	Homolog of Ly49A in 129 mice, Ly49D in C57L	129 Strain H2-D^k^, H2-D^b^, H2-D^d^, H2-L^d^ (Makrigiannis et al., 2001)
Ly49P	Ly49p1 = Arg; Ly49p2 = ψ; Ly49p3 = Arg	Klra16	p1 and p3 are **Activators**, p2 is **pseudogene** in NOD. Three genes in NOD mice, only one in 129 mice (Ly49P)	Ly49x (not same gene)	H2-D^k^; MCMV m04 protein (Kielczewska et al., 2009); 129 strain, weakly to H2-D^d^, H2-K^k^, H2-L^d^ (Makrigiannis et al., 2001)
Ly49Q	Ly49Q	Klra17	**Inhibitory**. Balb/c, NOD, B6, and 129; q2 and q3 are ψ in 129		H2-K^b^ (Scarpellino et al., 2007)
Ly49R		Klra18	**Activator**. 129 only	MGC130549; Homolog of Ly49D in 129 mice	129 Strain H2-D^k^, H2-D^b^, H2-D^d^, H2-L^d^ (Makrigiannis et al., 2001)
Ly49S		Klra19	**Inhibitor**, has unusual ITIM (Makrigiannis et al., 2001) 129 only		129 Strain weakly to H2-K^k^ (Makrigiannis et al., 2001)
Ly49T		Klra20	**Inhibitor**. 129 only		129 Strain did not bind to any H2 tested (Makrigiannis et al., 2001)
Ly49U	Ly49U	Klra21	**Activator**. NOD and 129 only	Homolog of Ly49H	129 Strain did not bind any H2 tested (Makrigiannis et al., 2001). Does not recognize MCMV m157 protein (Kielczewska et al., 2007)
Ly49u/i		Klra28 (see below) (Makrigiannis et al., 2002)	**Activator (putative)** 129 only	Klra11b; Ly49u/i^129^	Unknown
Ly49V		Klra22	**Inhibitory**. 129 only, genomic overlap with Klra15		129 Strain strongly to H2-D^k^, H2-D^b^, H2-D^d^, H2-L^d^ weakly to H2-K^b^, H2-K^d^, H2-K^k^ (Makrigiannis et al., 2001)
Ly49W	Ly49W	Klra23	**Activator**. NOD only	Ly49W1, Ly49W2	H2-L^d^, rat classical class I MHC molecule, RT1-A1^c^ (Lavender and Kane, 2006), H2-K^k^, H2-D^d^ (Dimasi and Biassoni, 2005)
Ly49X		Klra24	**Pseudogene** in NOD, and B6 mice		N/A
Ly49l/r	Ly49l/r	Klra25	**Activator (putative)** 129 only (Makrigiannis et al., 2002), not known if expressed		Unknown
Ly49Y			**Pseudogene** Balb/c only (Orr and Lanier, 2011)		N/A
		Klra26	**Pseudogene** 129 only	Ly49e/c1	Unknown
		Klra27	**Pseudogene** 129 only	Ly49e/c2	Unknown
		Klra28 (Makrigiannis et al., 2002)	**Activator (putative)** 129 only	Klra11b; Ly49u/i^129^	Unknown
		Klra14 (Klra29)	**Activator**. Predicted gene, homology with Klra8, Klra21	EG654449; OTTMUSG00000026959; Gm15858	Unknown
Ly49B		Klra30	**Activator**. B6, 129, Balb/c. Two isoforms (splice variants)	Ly49b, Klra2	See Ly49B above
		Klra31			Unknown
		Klra32		Homolog of Ly49D?	Unknown

**Table 2:** **Ly49 gene groups**.

A group (inhibitory)	a^B6, a^NOD^, a^Balb^, o^129^*^
G group (inhibitory)	t^129^, g_1–2_^NOD^, g^B6^, g^129^, g^Balb^
D group (activating)	d^B6^, d^NOD^, p_1–3_^NOD^, p^129^, pd^129^, pd_1–2_^NOD^, x^B6^, x^NOD^, r^129^
L group (activating)	l^Balb^, w^NOD^, m^B6^, m^NOD^
C group (inhibitory)	i_1–2_^NOD^, i_1_^129^, i^B6^, i^Balb^, c^NOD^, c^Balb/c^, c^B6^
E group (inhibitory)	e^NOD^, e^B6^, e^129^, e^Balb^, f^NOD^, f^B6^, s^129^**
H group (activating)	n^B6^, k^B6^, h^B6^, h^NOD^, u^NOD^, u^129^
Q group (inhibitory)	q_1_^129^, q^NOD^, q^B6^, q^Balb/c^
B group (inhibitory)	b^129^, b^B6^, b^Balb/c^

*Based on homology, genomic organization, and function (Belanger et al., 2008), groups of Ly49 genes can be clustered as above*.

There are two other aspects of Ly49 genes that add substantial complexity to their classification. Most surprisingly, different mouse strains have different numbers of the Ly49 genes encoded in the genome. The Makrigiannis lab has provided the best analyses of the genomics of the Ly49 genes so far and they found that some mouse strains, like Balb/c, have relatively few Ly49 genes with just nine, while the Non-obese Diabetic Mouse (NOD/ShiLtJ or NOD) strain has the most known to date with 22 Ly49 gene segments, nine of which are pseudogenes (Belanger et al., 2008). Additionally, the same genes vary in homology from strain to strain (Table 2; Figure 1). For example, Ly49E^Balb/c^ is homologous but not identical to Ly49E^NOD^. This diversity of the Ly49^NOD^ genes is very complex, due in part to duplications and pseudogenes (Figure 1). This is shown as a separate column in Table 1 and grouped into families in Table 2. Some of the genes have partial homology to two or more different genes and did not fit into the original nomenclature, for example, Ly49p/d1^NOD^ in Table 1, which shares partial homology with Ly49P at exon 3 and Ly49D at exon 4. The cloning process resulted in a few of these gene designations and were kept after complete sequencing to avoid further confusion, even if other exons showed homology to other Ly49 genes (Makrigiannis, personal communication).

Table 2 groups the genes into related families and updates data from a dendrogram in the NOD mouse study (Belanger et al., 2008). We’ve expanded it to include Ly49B and two members not included in that study, Ly49S^129^ and Ly49O^129^. Furthermore, there have long been close relationships in homology and antigenicity within the families, establishing groups shown in the same color on Table 2 based on the branches of the dendrogram by Belanger et al. (2008). A prime example of a closely related family is Ly49C, Ly49F, Ly49I, and Ly49H, which are recognized by some of the same monoclonal antibodies like SW5E6 and 14B11 (Table 3). It is important to note that the monoclonal antibody made in one strain won’t recognize the alleles from that same strain. For example, SW5E6 was made in 129 and won’t recognize Ly49I^129^ (Makrigiannis, personal communication).

**Table 3:** **Expression and binding antibody clones**.

Ly49	Expression	Antibodies
Ly49A	NK, NK T (To et al., 2009), NK1.1+ γδ (Hara et al., 2001), subset NK1.1−/DX5−/CD3+ T (Ortaldo et al., 1998); subset of CD8+ Tregs (Kim et al., 2011)	12A8, A1 (B6), JR9-318 (B6, Balb/c); YE1/32, YE1/48 (Held et al., 1995)
Ly49B	CD11b+/F4/80+/Gr1+ myeloid cells, activated NK, iNKT cell line, granulocytes (neutrophils, eosinophils, mast cells) macrophages, independent of Ly49Q (Held et al., 1995; Gays et al., 2006)	1A1 (Gays et al., 2006)
Ly49C	NK, NK T (To et al., 2009), NK1.1+ γδ (Hara et al., 2001), subset NK1.1−/DX5−/CD3+ T (Ortaldo et al., 1998); subset of CD8+ Tregs (Kim et al., 2011)	Ly49C/I SW5E6; Ly49C/F/I/H^B6^ 14B11
Ly49D	NK only, not on DX5− T (Ortaldo et al., 1998)	4E5; 12A1
Ly49E	Fetal NK, thymic NKT (Aust et al., 2011), γδ T (Van Beneden et al., 2001)	Ly49E/C binds 4D12 (Van Beneden et al., 2002) CM4 (Ly49E/F) (Aust et al., 2011)
Ly49F	Expression unclear (Corral et al., 1999); CD8+ Tregs (Kim et al., 2011)	Ly49C/I/H^B6^ 14B11
Ly49G	NK, NK T (To et al., 2009), NK1.1+ γδ (Hara et al., 2001), subset NK1.1−/DX5−/CD3+ T (Ortaldo et al., 1998); subset of CD8+ Tregs (Kim et al., 2011)	4D11; Ly49G2^B6^ Cwy-3; Ly49G^129/Balb^ AT8
Ly49H	NK, others unknown	14B11 which recognizes Ly49C/F/I (Corral et al., 1999)
Ly49I	NK, NK T (To et al., 2009), NK1.1+ γδ (Hara et al., 2001)	3D10; Ly49C/I SW5E6; Ly49C/I/F/H^B6^ 14B11
Ly49Q	Myeloid cells CD11b+/B220+/CD4+, and/or CD8+ (Toyama-Sorimachi et al., 2004); pDCs (Kamogawa-Schifter et al., 2005); and Osteoclasts (Hayashi et al., 2010)	
Ly49O	NK, others unknown	YE1/32, YE1/48 (Held et al., 1995)
Ly49V	NK	4E 5

There are 26 known rat Ly49 genes as well. The Ly49 genes in rat appear to be fairly distant from the mouse homologs, with Ly49i8 showing homology to mouse Ly49B, and mouse Ly49Q somewhat similar to a small cluster of 7 rat Ly49 genes from 26 functional genes. The rat gene cluster also has numerous pseudogenes (Nylenna et al., 2005).

## Ligands

Many Ly49 genes recognize “self” ligands like the MHC Class I molecules H2-D, H2-L, and H2-K (Table 1) (Makrigiannis and Anderson, 2000; Makrigiannis et al., 2001; Dimasi and Biassoni, 2005; Lavender and Kane, 2006; Kielczewska et al., 2007; Scarpellino et al., 2007; Belanger et al., 2008; Jonsson et al., 2010). The inhibitory Ly49 receptors, like Ly49A, prevent killing upon recognition of their cognate ligand. This is not always strain specific recognition. For example, NK cells expressing Ly49A^B6^, which recognize H2-D^b^ and H2-K^b^ from B6 mice, can also recognize H2-L^d^ and H2-D^d^ which are expressed in other mouse strains (Johansson et al., 2009). The absence of these ligands or “missing self” on target cells combined with other activating signals to the NK lymphocyte triggers a cytolytic response by the NK cell against the target cells. The inhibitory Ly49 genes can recognize their ligands both in cis (on the same cell) and trans (on adjacent cells) (Back et al., 2009) which involves a molecular shift in the shapes of the molecules (Back et al., 2011). This has been modeled with Ly49A dimers interacting with pairs of H2-D molecules in trans via an open conformation of the NKD interacting with β2 microglobulin and the α3 domain of H2-D^d^ on adjacent cells. This sends inhibitory signals to the NK cell. In cis conformations, the NKD dimers of Ly49C can interact with one H2-K^b^ in a closed conformation and, by inhibiting this inhibitory molecule, decreasing the activation threshold of the NK cell (Dam et al., 2003; Held and Mariuzza, 2008; Back et al., 2009, 2011).

Many activating Ly49 receptors also bind to MHC Class I molecules (Dimasi and Biassoni, 2005), but this is primarily recognition of different strain alleles or “non-self/altered-self” MHC molecules, and is defined using allogenic cytotoxic assays used to characterize the function of these receptors *in vitro*. In addition to recognition of mouse surface proteins, Ly49W^NOD^ and Ly49G^Balb/c^ can xenogenically recognize the rat class I MHC molecule, RT1-A1^c^ (Lavender and Kane, 2006). Ly49D^B6^ can be activated by Chinese hamster ovary (CHO) cells by a molecule mapped to the chok locus. The closely related C57L strain of mice also seems to interact with CHO cells using Ly49O^C57L^ which is very homologous to Ly49D^B6^ and reacts to the 4E4 antibody, yet is an inhibitory Ly49 (Mehta et al., 2001).

There is substantial evidence for tolerance and restriction by self MHC Class I molecules and regulating development (“licensing”) of NK cells via specific Ly49 genes (Lowin-Kropf et al., 2000; Kim et al., 2005). Studies using NK cells expressing single Ly49 molecules combined with transgenic expression of single MHC Class I ligands showed that Ly49 binding is not restricted as originally thought. Instead, many Ly49 molecules can recognize several different MHC class I molecules (Brodin et al., 2012). Belanger et al. (2008) have also used transgenic mice in which the Ly49O gene was targeted. They discovered that disruption of Ly49O unintentionally lowered transcription of all Ly49 genes. NK cells from these Ly49-knockdown mice were tested for killing against cells expressing or lacking MHC Class I genes *in vivo* and *in vitro*. It was found that killing of cells lacking MHC Class I was impaired by NK cells from these mice and could be rescued by restoring Ly49I expression, which is a strong inhibitory receptor for both H-2K^b^ and H-2D^b^, but not by restoring Ly49A or Ly49G (Cheng et al., 2008).

Many viruses, such as those in the herpes family, inhibit surface expression of MHC Class I molecules as a way of preventing recognition by conventional cytotoxic CD8+, αβ T cell receptor+ T lymphocytes (Loch and Tampe, 2005). Although this allows the virus to hide in the cell from these classical T lymphocytes, infected cells are vulnerable to killing by NK cells. When activating Ly49 receptors recognize their viral ligands, such as the mouse herpes virus cytomegalovirus (MCMV) protein m157 by Ly49H, this triggers a cytolytic response in the NK cells against the infected cells (Dimasi and Biassoni, 2005). MCMV protein m157 is a ligand for Ly49H^B6^, Ly49H^NOD/^, and Ly49I^129^ (Arase et al., 2002). However, similar genes from different strains of mice, Ly49I^B6^ for example, do not recognize m157 (Tay et al., 1999; Kielczewska et al., 2007). Additionally, m157 variants can escape binding to Ly49H^B6^. Ly49C^B6^ and Ly49C^Balb^ also can bind to some m157 variants but these interactions do not appear to be critical for viral resistance (Corbett et al., 2011). Ly49H seems to be most critical for resistance to MCMV, but does not mediate resistance to herpes simplex virus 1 or ectromelia virus (Cheng et al., 2008). Another MCMV ligand for Ly49P is m04, which is a viral protein that may play a role in H2-D^k^ stabilization (Kielczewska et al., 2009). The development of memory-like NK cells, which expand, contract, and persist like other lymphocytes, has also been described to work, in part, via Ly49H activation via m157. This process takes on a similar “antigen” driven expansion phase which truly mimics that of the “classical” lymphocytes like CD8+ αβ T cell receptor+ lymphocytes (Sun et al., 2009).

The large number of Ly49 receptors lacking known ligands highlights another need in this field. There may be unconventional ligands for some of these receptors as well. For example, urokinase plasminogen activator has been identified as a ligand for Ly49E and it may play a role in wound healing or tissue growth (Van Den Broeck et al., 2008). It may be that some of the missing ligands may reveal novel functions for the Ly49 genes beyond the simple killing of infected cells, inasmuch as a role for NK cells in placental development has been uncovered via HLA-G and KIR2DL4/CD158d interactions in human pregnancy (Bryceson and Long, 2008).

## Cellular Expression of Ly49 Genes

Along with the lack of known ligands for many Ly49 genes, there is very limited characterization of cells expressing Ly49 (Table 3). Only 11 of the Ly49 receptors have monoclonal antibodies that recognize them. Further complicating the issue, many of the antibodies are cross-reactive for more than one Ly49 gene.

Most Ly49 genes are found on NK cells, invariant NKT (iNKT) lymphocytes, and γδ T lymphocytes. Two of the 11 known genes were expressed on cells other than NK cells, notably Ly49Q which is found on myeloid cells and plasmacytoid dendritic cell (pDCs) subsets (Kamogawa-Schifter et al., 2005), and plays a role in maturation of these pDCs (Toma-Hirano et al., 2009) and activation of osteoclasts (Hayashi et al., 2010). Ly49B also is found on CD11b+ myeloid cells including macrophages, granulocytes, and mast cells, and there has been one report of Ly49B on activated NK and an iNK T cell line. Expression on Ly49B and Ly49Q are on non-overlapping myeloid subsets, and can induce in NK cells (Gays et al., 2006). Subsets of CD8+ Tregs (as defined by CD44+, CD122+, and Qa-1 mediated suppression) express Ly49A, Ly49C/I (weakly), Ly49G2, but had substantial expression of Ly49F (Kim et al., 2011).

## Role of Ly49 in Infection

Some Ly49 genes are critical for defense against viral pathogens, particularly MCMV (Makrigiannis and Anderson, 2000). This fits into the paradigm of NK killing of cells that lack MHC Class I expression due to herpes virus interference by several viral proteins (Orr et al., 2005; Temme et al., 2010). Few studies have looked at the role of Ly49 genes in other infections and in other animal models. Depletion of cells expressing rat Ly49s3/s4/i3/i4 proteins in rats led to increased bacterial load after infection by *Listeria monocytogenes*. Furthermore, infection caused an increase in cells expressing several different Ly49 molecules (Shegarfi et al., 2009). Ly49G2 expression dominates early in this process in mice as well (Barao et al., 2011). A separate study looking at Ly49 genes after *Plasmodium yoelii* infection found a decrease of Ly49A, C/I, D, and G2 expressing cells in the spleen but an increase in the liver (Roland et al., 2006).

Our interest in the Ly49 genes came after studying host gene expression following infection by *Francisella tularensis* (Kingry et al., 2011). A hallmark of *Francisella* infection is the lack of a robust innate immune response thus allowing the bacterium to outpace host defenses and cause rapid disseminated infection (Hajjar et al., 2006; Bosio, 2011). The mouse model was used to track changes in host responses during infection with the live vaccine strain (LVS) as well as the highly pathogenic lab strain Schu4 as measured by full genome expression microarrays. We observed an overall decrease in expression of the Ly49G2 gene in the lungs of Schu4 infected mice, but increased expression of Ly49G2 at 120 h post infection in the lungs of LVS infected mice (Kingry et al., 2011). This may signify the ability of the highly virulent strain to evade host NK cells more efficiently than the LVS. Both the LVS and Schu4 strain caused widespread decreases in many other Ly49 genes in the spleen, particularly Ly49A, Ly49I, Ly 49H, and Ly49D. Ly49D expression in the spleen eventually increased following infection but only after extensive dissemination of both strains of the bacteria. Ly49A, D, and H are all activating receptors, while Ly49G and I are inhibitory receptors. These results are similar to what was observed with *Listeria* and *Plasmodium* species (Roland et al., 2006; Shegarfi et al., 2009). It is unclear why both activating and inhibitory receptors might be altered after infection but it may represent a route of host immune evasion by *Francisella* or changes in the activation potentials of NK and other Ly49 expressing cells responding to the infection. Together with the studies on MCMV infections, we suspect that these receptors are part of the host response to many different types of intracellular pathogens.

## Summary

Starting with the complex genetics of the Ly49 genes, there remain considerable gaps in our understanding of the precise and perhaps interactive roles of the receptors in the immune response. Even at a basic level, ligands and cellular expression patterns remain only partially deciphered. While the genes were originally linked to NK killing of tumors and recognition of viral infection, recent studies have shown roles for Ly49 on other cell types and with other functions in myeloid cells and the innate immune response. The likely requirements for these genes in combating intracellular viral, bacterial, and parasitic pathogens remain relatively unexplored as well. These intriguing results hint that there may be more functions of these receptors still to be discovered, especially as more is learned about the expression profiles and ligands of the Ly49 genes.

## Conflict of Interest Statement

The authors declare that the research was conducted in the absence of any commercial or financial relationships that could be construed as a potential conflict of interest.
